# The use of enamel matrix derivative for the treatment of the apically involved tooth: A case report

**DOI:** 10.1097/MD.0000000000018115

**Published:** 2019-11-27

**Authors:** Jun-Beom Park

**Affiliations:** Department of Periodontics, College of Medicine, The Catholic University of Korea, Seoul, 06591, Republic of Korea.

**Keywords:** alveolar bone loss, enamel matrix proteins, periodontal diseases, regeneration

## Abstract

**Introduction:**

The aim of this report is to present a case of an apically involved tooth with successful regeneration by only applying enamel matrix derivative. The root of the tooth was planed and the defect area was well debrided using various instruments, including curettes and an ultrasonic scaler, and the root surface of the tooth and the defect area were loaded with enamel matrix derivative.

**Patient concerns:**

A 32-year-old man visited the clinic due to a referral for the evaluation of his mandibular left first molar.

**Diagnosis:**

The clinical and radiographic assessment displayed the loss of the periodontium around the tested tooth with apical involvement of the mesial root. Bleeding upon probing was noted at the mandibular first molar, with the deepest periodontal probing depth of 15 mm.

**Interventions:**

A nonsurgical approach was firstly performed on the tooth, and the deepest probing depth was reduced to 12 mm. After re-evaluation, elevation of a full-thickness flap was done, the root of the tooth was planed, and the defect area was well debrided using various instruments, including curettes and an ultrasonic scaler. The defect area on the mandibular left first molar was grafted with enamel matrix derivative.

**Outcomes:**

The 7-month postoperative clinical and radiographic evaluation showed healthy gingiva and an increase in radiopacity. The final 1-year and 9-month postoperative evaluation showed that regeneration of bony defect was well maintained up to the final evaluation with reduction of probing depth.

**Conclusion:**

In conclusion, a case of apically involved tooth can be treated only with enamel matrix derivative after meticulous debridement with curettes and an ultrasonic scaler.

## 1 Introduction

Enamel matrix derivative was applied for enhancing periodontal regeneration including new cementum, alveolar bone, and periodontal ligament.^[1]^ A previous report demonstrated that enamel matrix derivative was superior to open flap debridement for the treatment of intrabony defects of the tooth.^[2]^ In wide intrabony defect, enamel matrix derivative is applied as a mixture with bone graft material for regeneration of damaged tissue.^[3]^ In a previous report, it was suggested that enamel matrix applied with bone graft material showed successful results for regenerating intrabony defects, and the effects were comparable to recombinant human platelet-derived growth factor-BB with bone graft material.^[4]^ The application and indications for enamel matrix derivative is on the constant increase. However, there are no known reports on the use of enamel matrix derivative for the improvement of clinical parameters in the treatment of an apically involved tooth. The aim of this report is to present a case of the apically involved tooth with successful regeneration by only applying enamel matrix derivative.

## 2 Ethics, consent, and permissions

The Institutional Review Board of the hospital provided approval for this study (KC18ZESI0750), and patient has provided informed consent for publication of the case.

## 3 Case presentation

**Figure 1:**
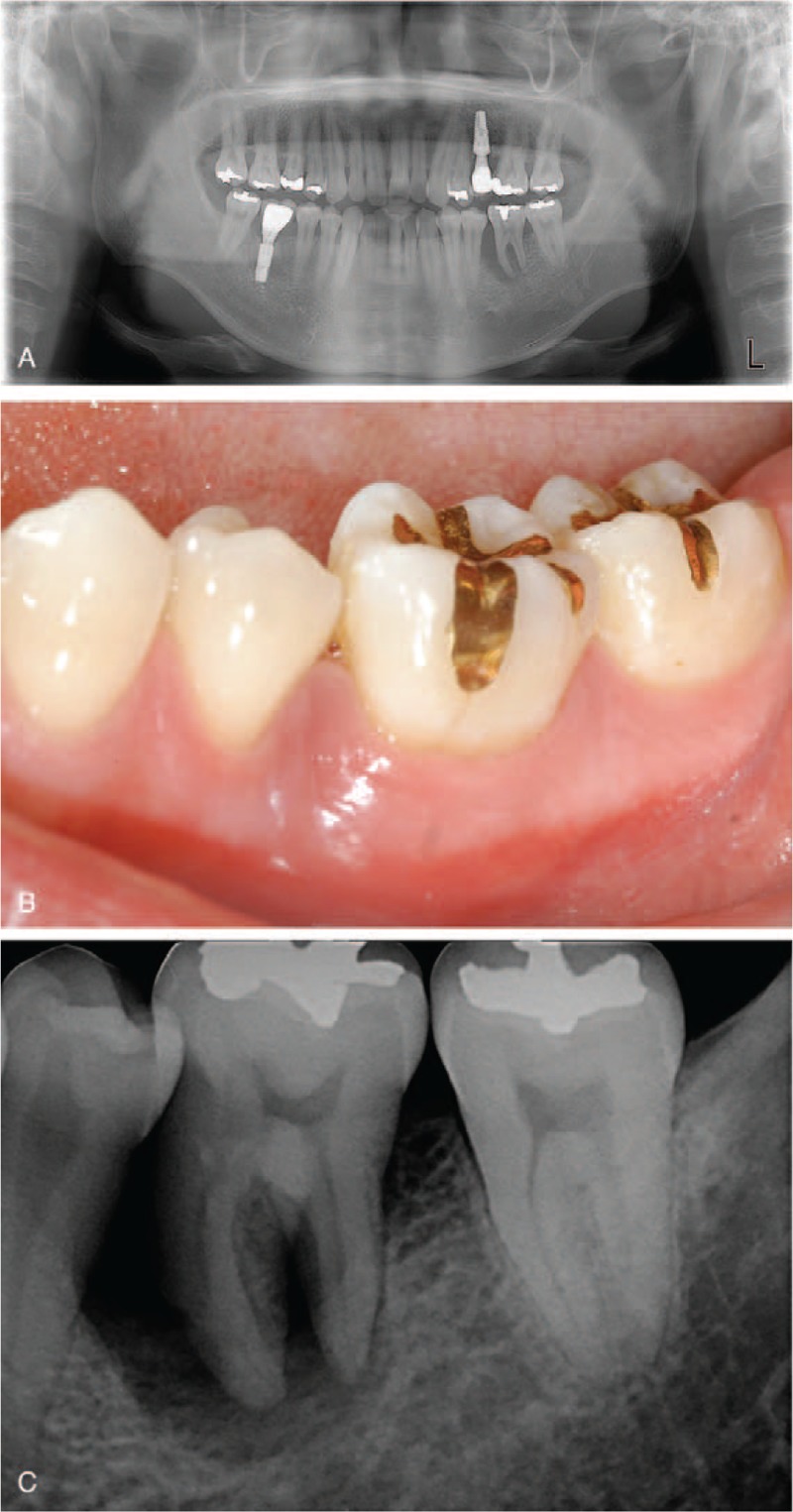
Evaluation of apically involved tooth. (A) Panoramic radiographic evaluation. (B) Buccal view showing gingival redness. (C) Periapical radiograph showing the loss of supporting bone around the tested tooth with apical involvement of the mesial root.

A 32-year-old male visited the Department of Periodontics due to referral for the evaluation of the mandibular left first molar. The patient had lost two teeth and had them replaced with dental implants (Fig. 1A). The patient had the intention to save the teeth if possible. Basic therapy included oral hygiene instructions and suggestions to refrain from possible habits including smoking and alcohol. Bleeding upon probing was noted at the mandibular first molar (Fig. 1B). The deepest probing depth of 15 mm was seen on the mesial side and at the mesial root. The radiographic evaluation showed the loss of periodontium around the tested tooth with apical involvement of the mesial root (Fig. 1C). The tooth was firstly treated with a nonsurgical approach, and the deepest probing depth was reduced to 12 mm. After re-evaluation, the area was planed for surgical treatment.

**Figure 2:**
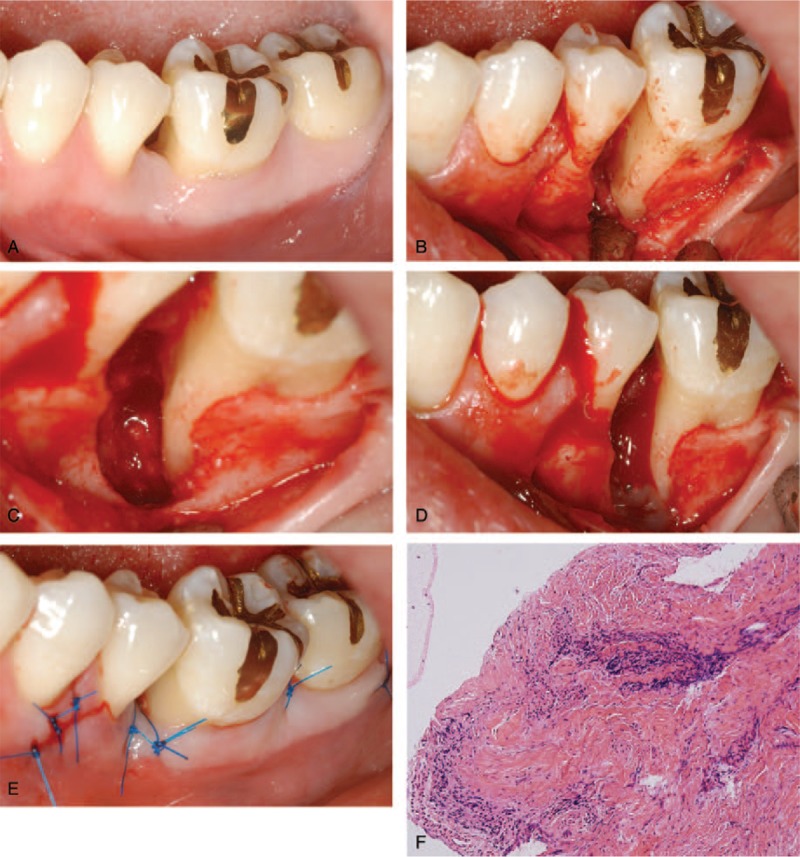
Surgical procedures. (A) Preoperative buccal view. (B) Buccal view after elevation of a full thickness flap showing loss of supporting bone. (C) The clinical photograph after meticulous debridement with curettes and an ultrasonic scaler. (D) Enamel matrix derivative was applied around the defect area. (E) The clinical photograph after application of sutures. (F) Histopathologic evaluation revealed chronic inflammation (hematoxylin-eosin stain; original magnification ×100).

The patient rinsed the intraoral area with 0.12% chlorhexidine digluconate solution (Hexamedine, Bukwang, Seoul, Korea) for 2 minutes before the periodontal surgery (Fig. 2A). Elevation of a full-thickness flap was done after injection of 2% lidocaine containing 1:100,000 epinephrine (Fig. 2B). There was a severe bony defect around the tooth, with severe loss of the buccal plate at the mesial root. The root of the tooth was planed, and the defect area was well debrided using various instruments, including curettes and an ultrasonic scaler (Satelec, Acteon, Merignac, France) (Fig. 2C). The defect had dimensions of the cementoenamel junction to the bony crest of 14 mm, bony crest to bony apex of 6 mm, and horizontal defect of 6 mm on the coronal side, with 4 mm in the apical area. The defect area was grafted with enamel matrix derivative (Emdogain; Straumann AG, Basel, Switzerland) (Fig. 2D). The flap was repositioned and was secured with nonabsorbable sutures (5-0, Ethicon, Johnson & Johnson, Somerville, NJ) (Fig. 2E). The biopsy was performed for the histopathologic analysis at the Department of Pathology, and the results showed chronic inflammation.

**Figure 3:**
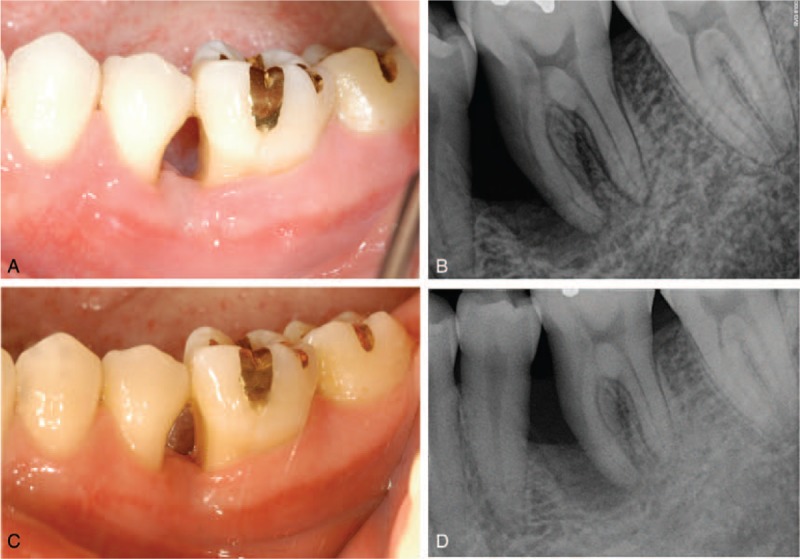
Postoperative follow-up. (A) Uneventful healing was achieved and sutures were removed 2 weeks after the operation. (B) Two-week postoperative periapical radiograph. (C) Two-month postoperative clinical photograph without symptoms. (D) Two-month postoperative periapical radiograph.

**Figure 4:**
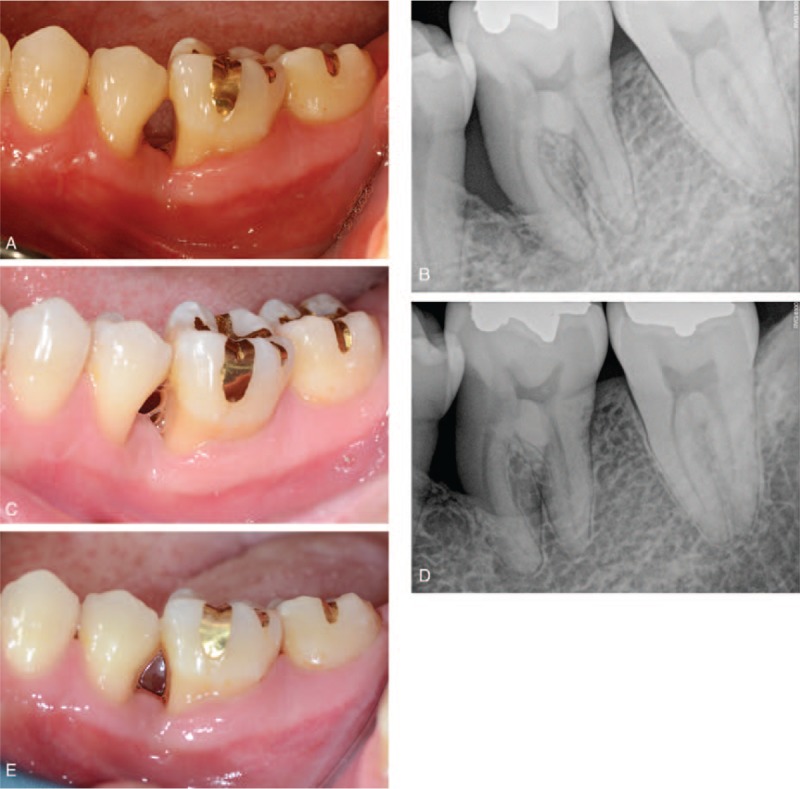
Follow-up check. (A) Seven-month postoperative buccal view showing healthy gingiva. (B) Seven-month postoperative radiograph showing increase in radiopacity. (C) One-year and 3-month postoperative clinical views indicating uneventful healing. (D) The radiograph at 1 year and 3 months postoperative, showing increased radiopacity around the left mandibular first molar. (E) Buccal view at 1 year and 9 months postoperative, showing healthy gingiva with regeneration of bony defect.

Uneventful healing was achieved and sutures were removed 2 weeks after the operation (Fig. 3A and B). A 2-month postoperative follow-up check was performed with eliminated symptoms (Fig. 3C and D). The 7-month postoperative clinical and radiographic evaluation showed healthy gingiva and an increase in radiopacity (Fig. 4A and B). The tooth was functioning well with stabilized radiopacity at 1-year and 3-month postoperative with deepest probing depth of 4 mm (Fig. 4C and D). Final evaluation at 1-year and 9-month postoperative showed that regeneration of the bony defect was well maintained (Fig. 4E).

## 4 Discussion

This report showed successful regeneration of the apically involved tooth with only enamel matrix derivative after meticulous debridement with curettes and an ultrasonic scaler.

Successful tissue regeneration has been achieved through the use of various materials.^[5,6]^ Enamel matrix derivative has been used for wider applications including soft tissue surgery and dental implants. The buccal plate extraction socket was regenerated with enamel matrix derivative and bone substitute.^[7]^ In more recent years, enamel matrix derivative was applied for the coverage of multiple gingival recession by applying a coronally advanced flap.^[8]^ A previous report stated that enamel matrix derivative showed clinically and esthetically satisfactory results on multiple recession defects with the modified tunnel technique.^[9]^ Successful treatment of palatal radicular groove-associated deep intrabony defects was done with the application enamel matrix derivative without endodontic treatment or retreatment.^[10]^ The use of enamel matrix derivative was suggested to be an effective means of periodontal regeneration in patients with rapidly advanced inflammatory process, leading to the destruction of periodontal tissue.^[11]^ In another report, the titanium implant surface was coated with enamel matrix derivative and faster soft tissue healing was shown with a larger quantity of soft tissue.^[12]^ Furthermore, peri-implantitis was treated successfully with enamel matrix derivative and bovine-derived hydroxyapatite.^[13]^

Enamel matrix derivative is shown to have osteoinductive properties.^[14]^ Application of enamel matrix derivative is reported to have higher gene expression in human bone cells regarding formation of extracellular matrix.^[15]^ Enamel matrix derivative is known to produce regenerative response in periodontal tissues and this response is partly replicated by amelogenin or ameloblastin components.^[16]^ Topical application of enamel matrix derivative on the soft tissues surrounding implants produced an increased number of blood vessels, which suggested beneficial effects on would healing.^[17]^ Moreover, enamel matrix derivative has been reported to increase angiogenesis by enhancing proliferation and migration of endothelial cells.^[18,19]^ Enamel matrix derivative was shown to significantly decrease expression of interleukin-1β and receptor activator of nuclear factor kappa-B ligand and increase expression of prostaglandin E2 and osteoprotegerin.^[19]^

In a previous report, one- to two-wall periodontal defects were treated with enamel matrix derivative alone or with bone substitute, and it was shown that the adjunct bone substitute with enamel matrix derivative improved the clinical and radiographic outcomes in unfavorable intrabony defects.^[20]^ However, it was suggested that enamel matrix derivative can be used alone in periodontal regeneration despite the limitation due to the gel-like consistency, especially in non-self-supporting defects.^[21]^ The results of previous report showed that the use of enamel matrix derivative alone and the use of a combination of enamel matrix derivative and deproteinized bovine bone mineral for the treatment of partially contained defects showed comparable clinical and radiographic outcomes after 12 months.^[22]^ The 2-year follow-up study evaluating the effects of enamel matrix derivative with particulate autogenous bone in the treatment of noncontained intrabony defects reported that all defects showed favorable clinical and radiographic outcomes.^[23]^ Similarly, this report demonstrated successful regeneration of an apically involved tooth with only enamel matrix derivative.

Enamel matrix derivative can be dissolved in propylene glycol alginate at an acidic pH, and the viscosity of enamel matrix derivative is decreased under physiologic conditions of neutral pH and body temperature, resulting in precipitation of enamel matrix derivative.^[24]^ It should be also noted that enamel matrix derivative is reported to remain at the site of application for up to 2 weeks.^[25]^ The reentry measurements of the intrabony defects treated with enamel matrix derivative after at least 5-year observational period showed stable results of complete resolution from five out of seven cases.^[26]^

In conclusion, a case of an apically involved tooth can be treated only with enamel matrix derivative after meticulous debridement with curettes and an ultrasonic scaler.

## Author contributions

**Conceptualization:** Jun-Beom Park.

**Data curation:** Jun-Beom Park.

**Investigation:** Jun-Beom Park.

**Methodology:** Jun-Beom Park.

**Resources:** Jun-Beom Park.

**Validation:** Jun-Beom Park.

**Writing – original draft:** Jun-Beom Park.

**Writing – review & editing:** Jun-Beom Park.
